# Sea Water and Menstruation

**Published:** 1897-03

**Authors:** 


### SEA WATER AND MENSTRUATION.

Dr. Houzel (*Ann. de Gynec.*) has published a series of statistics on menstruation in fishwomen on the French coast. They lead a hard life, and are ill-fed. They spend a great part of the year shrimping, which involves immersion for hours in sea water, often above the waist. They walk about in their wet clothes afterward, selling their shrimps. In winter they pick mussels out of rock pools at ebb tide, and return to town carrying baskets full of the mollusks, dripping over their clothes, the water sometimes freezing as it falls. All the 123 fisherwomen interrogated by Houzel insisted that the catamenia were always easier when they were actively at work at their calling. Some found that the period became painful or scanty when they led a temporarily dry life, and returned to its normal state when once more they walked in the sea to earn their bread. Puberty comes on rather earlier than in landswomen, the menopause later, and their fertility is markedly high. In short, fisherwomen are strong, and their period is retained at a normal point more steadily than is the case with other women. Houzel, however, sees a direct relation between the immersions and the normal catamenia. He notes that lady visitors, after a few days' acclimatization, find that sea bathing is excellent for regulating the catamenia. —*N. Y. Medical Times.*

